# Double Stranded Sperm DNA Breaks, Measured by Comet Assay, Are Associated with Unexplained Recurrent Miscarriage in Couples without a Female Factor

**DOI:** 10.1371/journal.pone.0044679

**Published:** 2012-09-17

**Authors:** Jordi Ribas-Maynou, Agustín García-Peiró, Alba Fernandez-Encinas, Maria José Amengual, Elena Prada, Pilar Cortés, Joaquima Navarro, Jordi Benet

**Affiliations:** 1 Càtedra de Recerca Eugin-UAB, Universitat Autònoma de Barcelona, Bellaterra, Spain; 2 Departament de Biologia Cel lular, Fisiologia i Immunologia, Universitat Autònoma de Barcelona, Bellaterra, Spain; 3 UDIAT, Centre Diagnòstic. Corporació Sanitària Parc Taulí. Sabadell. Institut Universitari Parc Taulí – UAB, Sabadell, Spain; 4 Servei de Ginecologia, Hospital Universitari Mútua de Terrassa, Terrassa, Spain; 5 Departament de Genética i Microbiologia, Universitat Autònoma de Barcelona, Bellaterra, Spain; University Hospital of Münster, Germany

## Abstract

It is known that sperm samples from recurrent pregnancy loss (RPL) couples have an increase in their sperm DNA fragmentation (SDF), but no studies have been performed in order to identify differences between single stranded SDF (ssSDF) and double stranded SDF (dsSDF) in these patients. This could be relevant because the type of DNA damage could have different effects. Semen samples were classified attending their clinical status: 25 fertile donors and 20 RPL patients with at least two unexplained first trimester miscarriages. SDF was analysed using alkaline and neutral Comet assay, SCD test and pulsed-field gel electrophoresis (PFGE), and ROC analysis including data from 105 more infertile patients (n = 150) was performed to establish predictive threshold values. SDF for alkaline and neutral Comet, and the SCD test was analysed in these categories of individuals. Data revealed the presence of two subgroups within fertile donors. The values obtained were 21.10±9.13, 23.35±10.45 and 12.31±4.31, respectively, for fertile donors with low values for both ssSDF and dsSDF; 27.86±12.64, 80.69±12.67 and 12.43±5.22, for fertile donors with low ssSDF and high dsSDF; and 33.61±15.50, 84.64±11.28 and 19.28±6.05, for unexplained RPL patients, also showing a low ssSDF and high dsSDF profile. This latter profile was seen in 85% of unexplained RPL and 33% of fertile donors, suggesting that it may be associated to a male risk factor for undergoing RPL. ROC analysis regarding recurrent miscarriage set the cut-off value at 77.50% of dsDNA SDF. PFGE for low ssSDF and high dsSDF profile samples and positive controls treated with DNase, to induce dsDNA breaks, showed a more intense band of about 48 kb, which fits the toroid model of DNA compaction in sperm, pointing out that some nuclease activity may be affecting their sperm DNA in RPL patients. This work identifies a very specific SDF profile related to the paternal risk of having RPL.

## Introduction

Recurrent pregnancy loss (RPL) is defined as having at least two consecutive embryo miscarriages within the first or early second trimester of pregnancy [1]. Due to the complex aetiology involved in miscarriages, up to 40%–50% of RPLs remain unexplained [1], [2]. Taking into account that sperm cells and oocytes provide half of the nuclear embryo DNA, it may be assumed that both males and females could be involved in either infertility or RPL [3], [4].

Regarding female factors that may be involved in RPL, they can be classified as genetic or chromosomal causes, advanced maternal age, antiphospholipid syndrome, hormonal abnormalities, uterine abnormalities or metabolic disorders [1], [2], [5]–[8]. The male factor has been less studied for many years, mainly basing the infertility diagnosis on semen parameters and, although this information is necessary, it is not always conclusive [9]. It has been described that the male factor may be involved in RPL when poor semen parameters, Y chromosome microdeletions, or a higher percentage of sperm aneuploidies detected by FISH are found [10]–[18]. However, normal sperm parameters are shown in many reported cases of RPL [17]. As a consequence, the paternal effect in these cases is being underestimated, and only a few recent reports provide data suggesting the possible relation of the sperm DNA status in the aetiology of RPL [12], [19]. Sperm DNA fragmentation has now become a new biomarker for male infertility diagnosis and different methods have been developed [20]–[26]. In fact, some studies have shown that sperm DNA fragmentation (SDF) is increased in semen samples from RPL couples by using Sperm Chromatin Dispersion test (SCD) [12], [15], Terminal deoxynucleotidyl transferase dUTP nick-end labelling (TUNEL) [11], [19] or Sperm Chromatin Structure Assay (SCSA) [10] methodologies. However, no studies have been performed analysing both single and double stranded DNA fragmentation in RPL patients. It has been recently reviewed that fertilisation with damaged spermatozoon may result in an increase of DNA damage in the embryo genome, which could result in DNA errors at different levels of embryogenesis [4], and it could end up as a miscarriage or different childhood diseases [27], [28].

**Figure 1:**
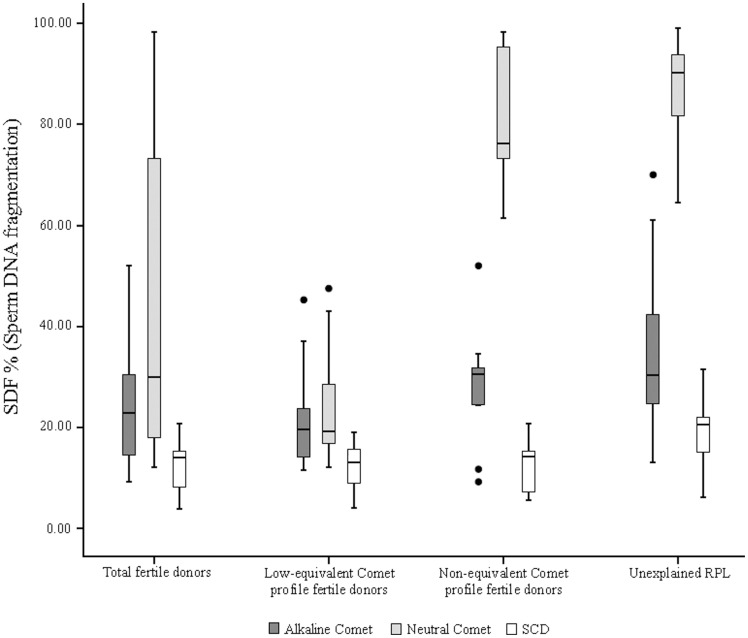
Alkaline Comet, neutral Comet and Sperm Chromatin Dispersion for total fertile donors, for low dsSDF and high dsSDF fertile donor subgroups and for the unexplained RPL group.

**Table 1:** Sperm DNA fragmentation (mean ± SD) in fertile donors and unexplained RPL samples.

	% SDF (Sperm DNA fragmentation)		
	Alkaline Comet	Neutral Comet	SCD
Total fertile donors (n = 25)	23.53±10.79	44.00±30.18	12.35±4.55
Low dsSDF fertile donors (n = 16)	21.10±9.13	23.35±10.45^c^	12.31±4.31
High dsSDF fertile donors (n = 9)	27.86±12.64	80.69±12.67a, b	12.43±5.22
Unexplained RPL (n = 20)	33.61±15.50a, b	84.64±11.28a, b	19.28±6.05a, b, c

a Statistical differences with total fertile donors (p<0.01).

b Statistical differences with low dsSDF fertile donors (p<0.01).

c Statistical differences with high dsSDF fertile donors (p<0.01).

The higher sperm DNA fragmentation found in previous works studying RPL patients may have its origin in poor DNA packaging, at chromatin remodelling during spermiogenesis, which could leave DNA more vulnerable basically in front of oxidative stress [29]–[32] and DNA nucleases [33], [34]. Some papers have described the sperm chromatin compaction showing the toroids as the basic structural elements separated by a linker DNA attached to the nuclear matrix, known as matrix attachment region (MAR), which would be more susceptible to being cut by nucleases [35]–[37]. Each toroid compacts about 48 kb of DNA, which represents a unique degree of DNA packaging in sperm [37]. Moreover, other authors showed the importance of chromosome organisation in the sperm nucleus, pointing out that centromeres might be grouped in internal regions of the sperm and telomeres would be associated in pairs at more outer layers [38]–[40].

In a previous study, alkaline Comet assay, identifying mostly single stranded DNA fragmentation (ssSDF), and neutral Comet assay, identifying mostly double stranded DNA fragmentation (dsSDF), were compared in controls and in different groups of patients [41], [42]. Different DNA damage profiles were found due to different aetiologies of DNA fragmentation in different infertile patients and chromosome reorganisation carriers [41]. Then, different single and double stranded DNA damage profiles were established: a) a profile with low percentages of sperm with both ssSDF and dsSDF, which has been seen in most fertile donors; b) a profile with low percentages of sperm with ssSDF and high percentages of dsSDF, which was seen in chromosome reorganisation carriers and three fertile donors [41] and with still unknown consequences on fertility and c) a profile with both high percentages of ssSDF and dsSDF, which has been shown in varicocele patients [41] and linked to the worst prognosis for fertility.

The aim of the present work is to describe the single and double stranded DNA fragmentation, by using alkaline and neutral Comet and SCD test, in semen samples from RPL couples without female factors. Then, to establish different threshold values for both pregnancy and recurrent miscarriage, and additionally, to improve the knowledge of the causes and the possible localisation of these dsDNA breaks by using pulsed-field gel electrophoresis (PFGE).

**Figure 2:**
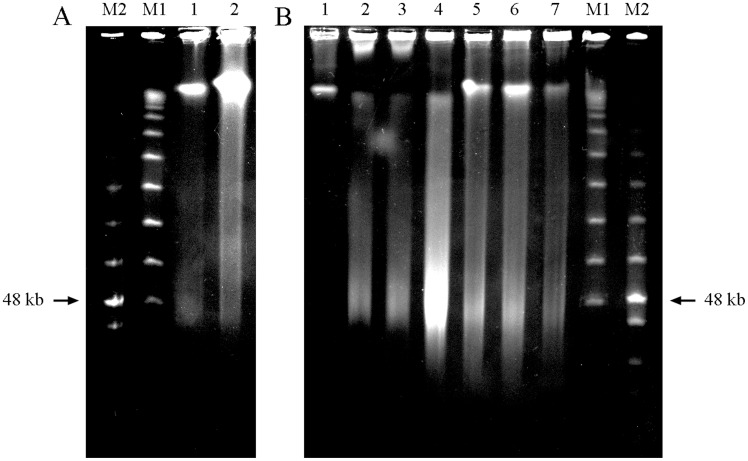
Pulsed-field gel electrophoresis of semen samples DNA from fertile donors (A, lanes 1 and 2; B, lane 1), negative control (B, lane 1), positive controls with DNAse 0.5 mg/ml, 30 minutes (B, lanes 2, 3 and 4) and RPL samples (B, lanes 5, 6 and 7). DNA molecular weight markers consisting of Low Range PFG Marker (M1) and Lambda ladder PFG marker (M2) are detailed. Negative controls in B, lane 1 show a thin compression zone. Positive controls in B, lanes 2, 3 and 4 show DNA digestion into sizes of around the 48 Kb. Sperm DNA fragmentation of the specific samples of this figure is shown in Table 2.

**Table 2:** Relation of samples shown in Figure 2 with their sperm DNA fragmentation.

% SDF		A		B						
	Lane	1	2	1	2	3	4	5	6	7
Alkaline Comet(ssSDF)		11.75	24.5	14.6	–	–	–	30.4	24.2	21.25
Neutral Comet(dsSDF)		98.25	95.75	18.0	–	–	–	96.6	90.0	94.75

## Materials and Methods

### Semen Samples

Semen samples from 45 human males were obtained in collaboration with reproduction centres of the Barcelona area and were divided into two groups: 25 donors with proven fertility and without experiencing any previous miscarriage (15 previously reported, [41]) and 20 donors from couples with at least two consecutive miscarriages within the first or early second trimester of pregnancy. In the RPL samples, abnormal female factors for advanced maternal age, karyotype, antiphospholipid antibodies, uterine abnormalities and thrombophilias were discarded.

Samples were obtained by masturbation after a minimum of three days of abstinence. A semenogram was performed according to WHO 2010 and samples were cryopreserved in test-yolk buffer (14% glycerol, 30% egg yolk, 1.98% glucose, 1.72% sodium citrate) [41], [43]. Sperm count (spermatozoa/mL), motility (A+B %) and morphology (Kruger strict criteria, normal forms %) were (mean ± standard deviation): 109.88±114.54, 37.20±23.02 and 7.20±1.87, respectively, for the fertile donor group and 116.65±115.83, 39.18±19.44 and 5.00±2.45, respectively, for the RPL group.

Informed consent was obtained from all donors and the present study was approved by the appropiate ethics committee.

**Figure 3:**
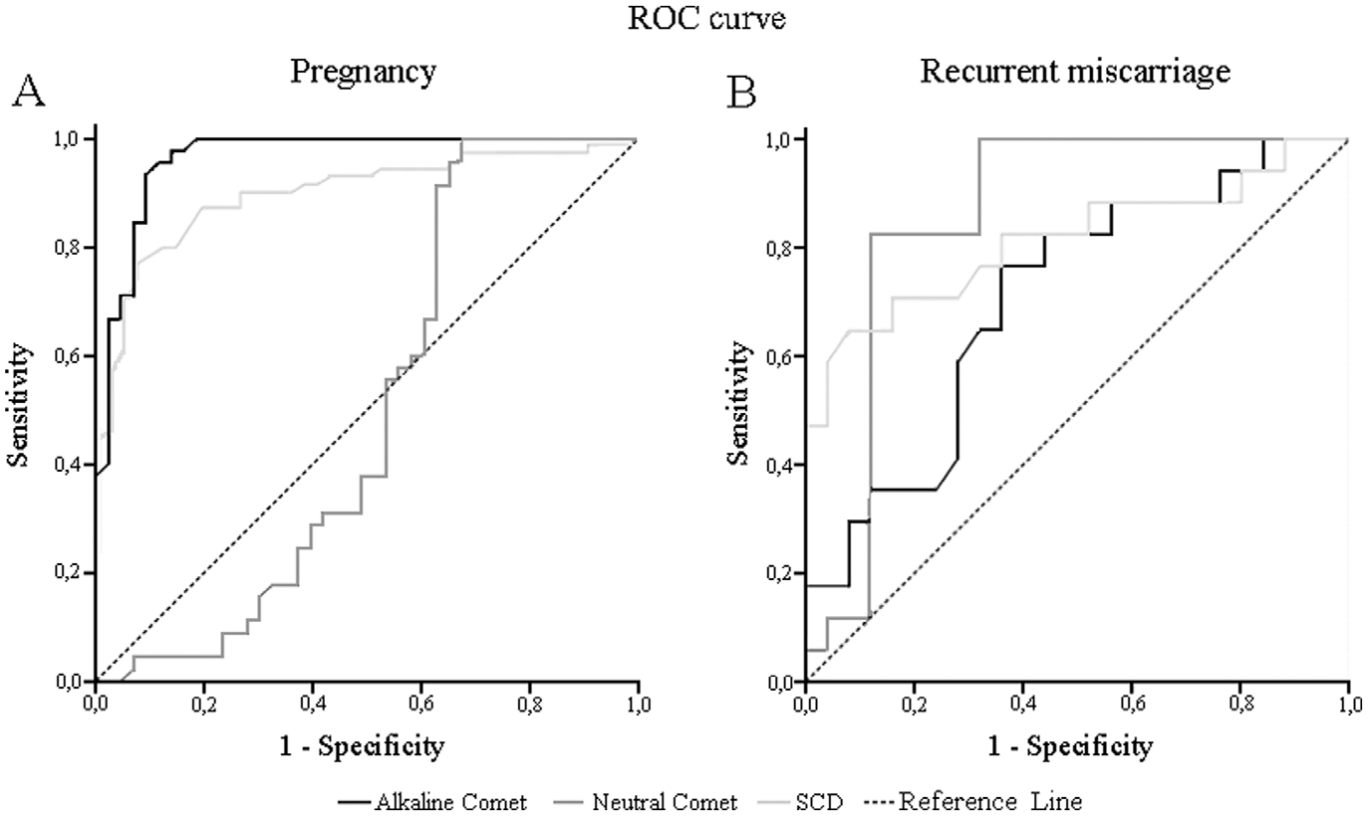
ROC curves analysis for alkaline Comet, neutral Comet and SCD attending: Pregnancy without miscarriage (A), and recurrent miscarriage (B).

### Neutral and Alkaline Comet Assay

Alkaline and neutral Comet assay procedures, staining and classification of fragmented or non-fragmented sperm were performed on all semen samples according to the protocol reported before [41]. Intra-individual differences were measured in five samples and the variability mean was less than 5% of SDF for both alkaline and neutral Comet. These results have been previously published [41].

Alkaline and neutral Comet assays were perfomed simultaneously in two different slides. First, an aliquot of the total semen was thawed and washed three times in PBS. Then, sperm cells were diluted to a concentration of 10×10^6^ spermatozoa/ml, and 25 **µ**l were mixed with 50 **µ**l of low melting point agarose 1% (Sigma Aldrich; St Louis, MO, USA) in distilled water. Rapidly, 15 **µ**l of the mixture were placed on two different pre-treated slides for gel adhesion (1% low melting point agarose), covered with coverslips and allowed to jellify on a cold plate at 4°C for 5 minutes. Next, coverslips were carefully removed and slides were submerged for 30 minutes in two lysing solutions (Comet lysis solutions, Halotech; Madrid, Spain) and washed for 10 minutes in TBE (0.445 M Tris-HCl, 0.445 M Boric acid, 0.01 M EDTA). For the neutral Comet assay, electrophoresis was performed in TBE buffer at 20 V (1 V/cm) for 12 minutes and 30 seconds, and then washed in 0.9% NaCl for 2 minutes. For the alkaline Comet assay, the slide was incubated in denaturing solution (0.03 M NaOH, 1 M NaCl) for 2 minutes and 30 seconds at 4°C, and afterwards, electrophoresis was then performed in 0.03 M NaOH buffer at 20 V (1 V/cm) for 4 minutes. After that, both neutral and alkaline slides were incubated in the neutralizing solution (0.4 M Tris-HCl, pH 7.5) for 5 minutes, in TBE for 2 minutes and finally dehydrated in an ethanol series of 70%, 90% and 100% for 2 minutes each.

**Figure 4:**
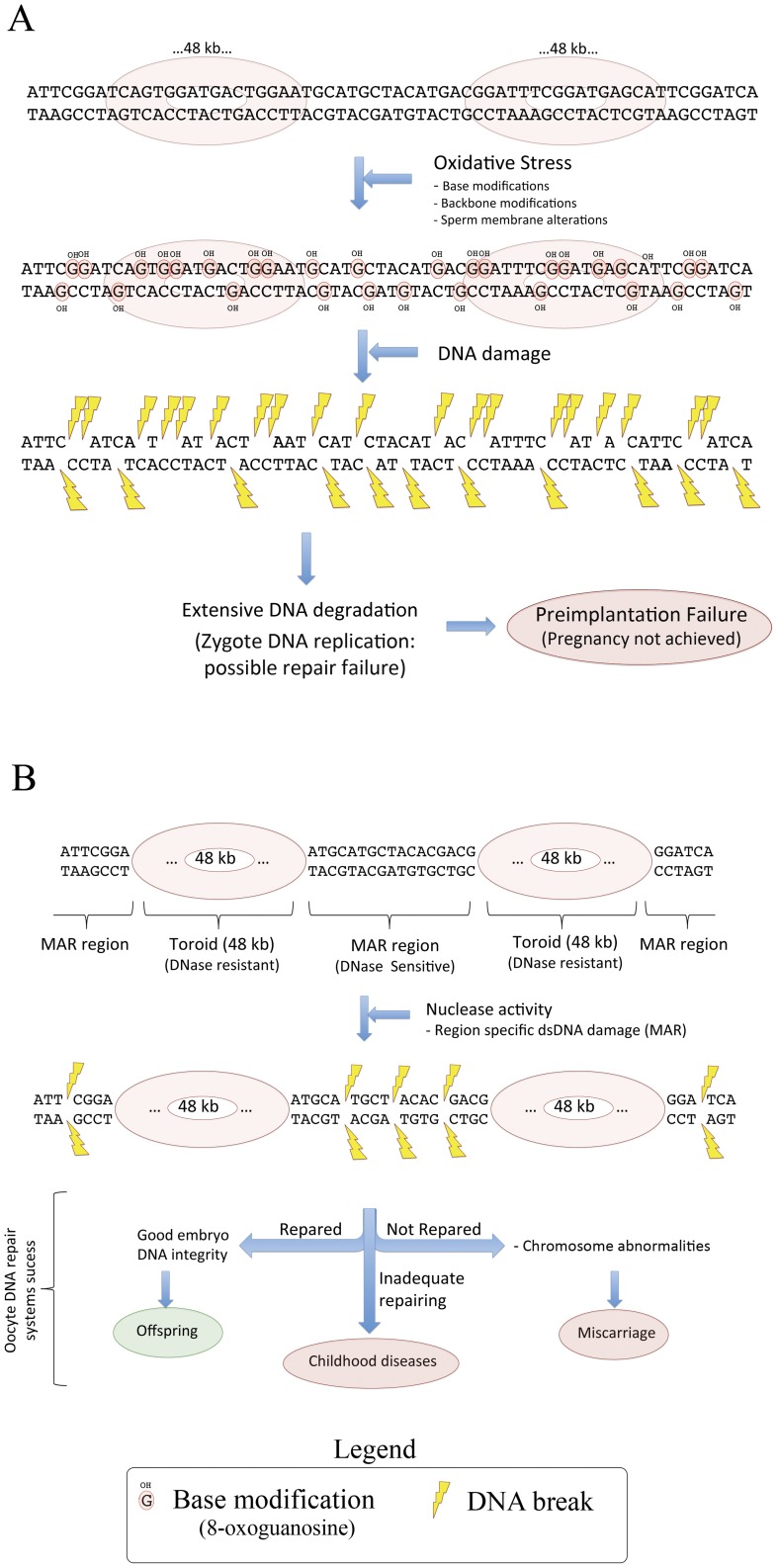
Model for ssDNA and dsDNA breaks mechanisms and clinical outcomes. ssSDF model (A) dsSDF model (B).

### Sperm Chromatin Dispersion Test (SCD)

Sperm DNA damage using the SCD test was performed using the Halosperm kit (Halotech DNA; Madrid, Spain) following the manufacturer’s instructions. Samples were stained with DAPI SlowFade® Gold antifade (Invitrogen; Eugene, OR, USA) and 400 spermatozoa were assessed and classified as fragmented or non-fragmented sperm, according to the manufacturer’s criteria, using a fluorescence microscope (Olympus AX70).

### Pulsed-field Gel Electrophoresis (PFGE) Method

23 of 45 samples from all the groups were analysed through PFGE in order to find size patterns of the DNA fragments.

#### Negative and positive controls

Sperm samples with a known profile of both low values of ssSDF and dsSDF were considered to be negative controls. Positive controls were induced using the same Comet assay profile samples, but with the following procedure: after thawing on ice and being washed twice in PBS for 2 minutes, sperm cells were centrifuged at 700 g to achieve a concentration between 15·10^6^ and 30·10^6^ spermatozoa per 100 µl. Then, sperm cells were permeabilised with 0.25% Triton X100 for 2 minutes on ice and two more washings in TBE 0.5X were performed. After that, in order to produce dsDNA breaks, a treatment with 0.5 µg/mL ribonuclease I from bovine pancreas (Sigma; St Louis, MO, USA) was performed for 30 minutes at 37°C and nuclease action was stopped with 50 mM EDTA. PFGE protocol was continued making the PFGE plugs as following described immediately below.

#### PFGE analysis

The pulsed-field gel electrophoresis protocol applied was similar to the protocol reported before [36]. Sperm cells were concentrated at 15–30 million spermatozoa in 100 µl and mixed with 1% 100 µl pulsed-field certified agarose (BioRad; Hercules, CA, USA), poured into insert moulds and allowed to solidify. For lysis, the resulting plugs were placed in 2 ml of lysis buffer (10 mM Tris HCl, 10 mM EDTA, 100 mM NaCl, 20 mM DTT, 2% SDS and 20 µg/mL proteinase K, pH 8.0) and incubated for 24 h at 53°C. The plugs were washed three times in TE+Glycine (10 mM Tris-HCl, 0.1 mM EDTA, pH 8 and 1 M Glycine) for 10 minutes, and then twice more in TE buffer for 10 minutes.

A quarter slice of each plug was cut off and placed on 1% gel and resolved by electrophoresis on a contour-clamped homogeneous electric field apparatus (Bio-Rad CHEF DRIII system) in TBE 0.5 X (Tris-borate 50 mM, EDTA 0.1 mM) at a 120° angle, 14°C, 4 V/cm and with the following pulses: 6.7 tp 33.7 seconds for 27.1 hours.

DNA molecular weight markers consisting of Lambda ladder PFG marker and Low Range PFG Marker (New England Biolabs; Ipswich, MA, USA) were included in each electrophoretic run.

Gels were stained with ethidium bromide and visualised and photographed under ultraviolet light using the GelDoc System (BioRad; Hercules, CA, USA).

### Statistical Analysis

Statistical analysis of SDF data was performed using the Statistics Package for the Social Sciences software, version 17 (SPSS Inc.; Chicago, IL). The Mann-Whitney U test was used to compare samples, setting the confidence interval at 95%, and ROC analysis was performed, including previous data of 105 infertile patients from our group (n = 150) ([41] and Garcia-Peiró unpublished data) in order to obtain the sensitivity, specificity and the cut-off value for each test.

## Results

### Sperm DNA Fragmentation: Alkaline and Neutral Comet Assay and SCD Test

Of all 25 collected semen samples from fertile donors, 16 samples (64%) presented a profile with low values of both ssSDF and dsSDF and nine samples (36%) presented a profile with low values of ssSDF and high values of dsSDF. Regarding RPL study samples, 17 out of 20 (85%) showed low values of ssSDF and high values of dsSDF.

Results and statistical comparisons of data obtained by using both alkaline and neutral Comet assays and the SCD test are shown in Table 1 and Figure 1. No statistical differences were obtained for either alkaline or neutral Comet assays with the increase of 10 more samples in the fertile donor group, with respect to the previously published control group [41] (p>0.05). However, this enlargement of the previously reported control group allowed for the observation of a bimodal distribution is dsSDF, suggesting the presence of two subgroups within it. These two fertile donor subgroups, one with a low ssSDF and low dsSDF profile and the other with a low ssSDF and high dsSDF profile, showed statistical differences in neutral Comet (p<0.01), nevertheless, no statistical differences were found between them regarding alkaline Comet (p>0.05) or the SCD test (p>0.05) (Table 1).

On the other hand, statistical differences were found in all three techniques when comparing unexplained RPL group SDF with total fertile donors SDF (p<0.01). Regarding the RPL group and low dsSDF fertile donor group, differences were found between them for all three techniques analysed (p<0.01). No differences were found for ssSDF or dsSDF between RPL and high dsSDF fertile donor group (p>0.05), however, statistical differences were found by using the SCD test (p<0.01).

### Pulsed-field Gel Electrophoresis (PFGE)

PFGE showed good reproducibility regarding the bands shown within the sample groups analysed. A PFGE analysis on sperm DNA is shown in Figure 2 as an example, and the relationship with the SDF of the samples analysed in that gel is shown in Table 2.

#### Negative and positive controls

The negative control obtained from a sample with a known low SDF for both ssDNA and dsDNA is shown in Figure 2B, lane 1, and shows a thin compression zone. Positive controls made using the same sample with incubations of DNAse to induce dsDNA breaks (Fig. 2B lanes 2, 3 and 4) showed DNA digestion into sizes of around 48 Kb.

#### Samples from fertile donors and RPL patients

Samples from three fertile donors are shown in: Figure 2A lanes 1 and 2, for samples with a low ssSDF and high dsSDF profile; and in Figure 2B, lane 1, for a sample with both low ssSDF and dsSDF. Samples with a low ssSDF and high dsSDF profile showed a slight compression band and also a band at about 48 kb, similar to positive controls with nuclease (Figure 2B; lanes 2, 3 and 4). The fertile donor shown in Figure 2B, lane 1, with both a low ssSDF and dsSDF, presented a compression band with good DNA integrity, and no 48 kb band was seen.

Results from RPL samples are shown in Figure 2B, lanes 5, 6 and 7. Both the thin compression band and the 48 kb sized fragments were present in these patients.

### ROC Analysis

ROC analysis results are shown in Figure 3, for either achieving a pregnancy without taking into account a possible subsequent miscarriage, and for undergoing a recurrent miscarriage associated with a male factor and without the female factors mentioned previously. Regarding the achievement of a pregnancy in all three techniques, ROC analysis set the cut-off value at 45.62% of alkaline Comet SDF, with a sensitivity and specificity of 0.933 and 0.907, respectively, and an area below the curve of 0.965 cm^2^. SCD data showed a cut-off value of 22.5% of SDF with a sensitivity and specificity of 0.768 and 0.929, respectively, and an area below the curve of 0.899 cm^2^. Neutral Comet showed lower combined sensitivity and specificity and less area below the curve in predicting pregnancy: 0.911, 0.349, 0.503 cm^2^, respectively (Figure 3A). Otherwise, regarding male-factor associated recurrent miscarriage, neutral Comet assay set the threshold value at 77.5% of SDF, with a sensitivity and specificity of 0.833 and 0.880, respectively, and an area below the curve of 0.858 cm^2^. SCD established the cut-off value at 18.5% of SDF, with a sensitivity and specificity of 0.647 and 0.920, respectively, and an area below the curve of 0.814 cm^2^. Alkaline Comet showed lower combined sensitivity and specificity and less area below the curve in predicting recurrent miscarriage: 0.944, 0.057, 0.303, respectively (Figure 3B).

## Discussion

Measurement of sperm DNA fragmentation is an area of growing interest due to its capacity of predicting male infertility [4], [44]–[46]. In a previous paper a descriptive study was performed on different groups of patients discussing the relationship about the different profiles of alkaline and neutral Comet assay regarding the aetiology of DNA breaks [41]. In the present work, ssSDF and dsSDF have been analysed in fertile donors group and RPL patients by using alkaline and neutral Comet assay, the SCD test and PFGE. Regarding fertile donors, a bimodal distribution has been observed in neutral Comet assay SDF, corresponding to dsDNA breaks, suggesting that two different subgroups could be identified within them: fertile donors with low ssSDF and low dsSDF, and fertile donors with low ssSDF and high dsSDF (Figure 1 and Table 1). These results point out that dsDNA breaks would not have implications on the achievement of a pregnancy. Low values of alkaline Comet assay SDF (<52%) are shown in both subgroups of fertile donors, showing its importance in achieving a pregnancy, as has been proposed recently for native semen using ART [47], and most of them showed a lower SDF than the 25% threshold value for natural conception [47].

About 85% of unexplained RPL patients included in the study showed low values of ssSDF and high values of dsSDF, and no differences were found when comparing them with the high dsSDF fertile donors group (Figure 1 and Table 1). Otherwise, statistical differences were found for both alkaline and neutral Comet assays upon comparing them with the low dsSDF fertile donors. However, alkaline Comet assay always showed values below the 52% threshold value established for the achievement of a pregnancy [47]. These Comet assay profiles applied to fertile donors and unexplained RPLs are consistent with previous reports because they were compatible with pregnancy by having a low percentage of single stranded DNA damage [47] and also with the fact that dsSDF might be a quality biomarker in sperm, that could be indicative about the progressive embryonic development. About that, some unknown parameter related to the oocyte capacity of repairing these double stranded DNA damage presented by the fertilising sperm could be important for appropriate **embryonic development (** **Figure** 1) [48]. In this sense, while a profile with low values for both ssSDF and dsSDF would mean a good prognosis of pregnancy and offspring, the profile with low ssSDF and high dsSDF would indicate a good prognosis for pregnancy, but with a risk of undergoing a male-factor associated miscarriage. It has also been described that fertilisation with damaged sperm could lead to errors in DNA replication, transcription and translation [4] because of the differential repair of single or double stranded breaks. For that, the distinction of ssDNA and dsDNA breaks seems to have an interest in the male factor diagnosis area, and the knowledge of the DNA breaks aetiology could provide new clues to understanding part of idiopathic RPL. Moreover, the sperm DNA damage assessment could be especially interesting in those patients with normal semen parameters, who are classified as idiopathic infertility.

The analysis of SDF by the SCD test showed no statistical differences between the two different fertile donor groups and, in consequence, the SCD test would not have the ability of distinguishing the high percentage of double stranded DNA breaks presented by the fertile group with high dsSDF found by the neutral Comet assay. The unexplained RPL group presented higher levels of SDF, when compared with the two fertile donor groups, in agreement with previous studies [11], [12], [15]. However, RPL samples presented a SDF at about the threshold value required for this method (20%–30%) [24] for achieving a pregnancy.

Data obtained with both Comet assay and the SCD test allowed for the establishment of different threshold values for each SDF technique for fertilisation success and miscarriage prognosis. Results displayed a threshold value of 45.62% SDF for the alkaline Comet assay related to pregnancy achievement. This result is in agreement with the 52% threshold for ART fertility proposed by Simon et al. [47], taking into account that this study did not differentiate natural conception and fertilisation after ART treatment, and that the Comet assay protocol used was slightly different [41], [42]. Regarding the SCD test, different threshold values have been proposed for achieving pregnancy [23], [49], and our analysis obtained a similar threshold value of 22.5% of SDF. Although both techniques can distinguish between fertile and infertile donors, alkaline Comet assay demonstrated higher sensitivity and specificity than the SCD test, in relation to fertility. Otherwise, neutral Comet assay, evaluating dsDNA breaks incidence, had no association with pregnancy achievement (Figure 3A). This lack of association with pregnancy might be due to the different oocyte repair mechanisms (Figure 4). Single stranded DNA damage is produced mainly due to oxidative stress, which induces base modifications, DNA backbone modifications and membrane alterations [50]. This DNA damage is extensive, being produced both in the MAR regions and within the DNA compacted by toroids, and could even be stronger if a bad DNA compaction is present. This extensive DNA damage finally produces a high number of DNA breaks and, because the ssDNA breaks are being repaired during the first embryo DNA replication [51], the presence of such extensive damage would make it difficult to be all repaired in the first embryo cleavage. This lack of repair due to this extensive damage would cause, in the end, a failed pregnancy. On the other hand, double stranded DNA damage is produced mainly due to nuclease activity, which directly produces DNA breaks in unprotected regions (MAR regions that are not compacted by protamines) [37] (Figure 2B). In consequence, this dsDNA damage is not as much extensive as ssDNA damage, and must be repaired before the replacement of protamines by histones in the embryo. There are three possible scenarios about the final outcome: a) If the dsDNA damage is not repaired by the embryo, it would cause chromosome abnormalities that would end up as a miscarriage; b) If the dsDNA breaks are repaired, then DNA integrity is recovered and the pregnancy and posterior birth can be carried out, and c) If dsDNA breaks have an inadequate repair, then there would be a few DNA alterations that could lead to childhood diseases (Figure 4). In this sense, our results show that the neutral Comet assay (dsSDF) had a good association with the male-factor associated miscarriage risk, induced by sperm DNA damage, with a threshold value of 77.5% of SDF and an acceptable sensitivity (0.833) and specificity (0.880) to be used as a diagnostic tool. For predicting the male-factor associated miscarriage risk, the SCD test established a threshold of 18.5%, but with lower sensitivity than neutral Comet assay. Otherwise, the alkaline Comet assay did not have any association with recurrent miscarriage, being the worst of the three techniques in RPL prognosis (Figure 3B). As the effect of ssSDF and dsSDF could have different implications in reproduction, our data suggest that semen samples need to be analysed with both alkaline and neutral Comet assay in order to obtain an accurate diagnosis. First, the alkaline Comet assay threshold of 52% would indicate the fertilisation capacity of the sample. Then, if neutral Comet is higher than 77.5%, the low ssSDF and high dsSDF profile shown would indicate the possibility of suffering a miscarriage, depending on the oocyte capacity of repairing the double stranded sperm DNA breaks. In fact, it has been demonstrated that better outcomes are obtained when oocytes from donors are used, compared with standard IVF cycles [52]. The combination of the two Comet techniques could also improve the global sensitivity and specificity of predicting a pregnancy, which could result in miscarriage.

Finally, regarding the possible origin of the dsDNA breaks shown by neutral Comet assay, it has been previously described the existence of some nuclease activity in sperm cells [36] whose activation should be linked to oxidative stress [53]. Both fertile donors with high dsSDF and unexplained RPL showed low values of oxidative damage, which is detected by alkaline Comet assay (ssSDF) [41] and high values of nuclease damage, which is detected by neutral Comet assay (dsSDF) [41], a reason that leads us to think that nuclease activity independent of oxidative stress should also exist. To confirm this approach, PFGE was performed on the different sample groups (Figure 2 and Table 2). Fertile donors with both low ssSDF and dsSDF showed a thin compression band (Figure 2B, lane 1), and incubations of this same sample with DNase, to induce dsDNA breaks, resulted in fragment sizes of around 48 kb (Figure 2B, lanes 2, 3 and 4). These results fit the toroid model of DNA compaction. Toroids of 48 kb are compacted on the sperm head, leaving a region of about two kilobases, the matrix attachment regions, which would be linked to the nuclear matrix, packaged by histones and, because of that, sensitive to nuclease activity [37]. DNases would not be able to cut toroid DNA compacted by protamines, but MAR regions, which are linked to histones, would be exposed to their nuclease activity [35], [36]. Samples from fertile donors with high dsSDF and from unexplained RPL patients showed both bands, one compression band at a high number of kb and the other at 48 kb, similar to the band that appeared with DNase treatment, which would agree with the approach that some nuclease activity, independent of oxidative stress, affects the DNA of these donors and RPL patients. The compression band would have a relation with a low level of ssSDF, and the 48 kb band would be related to a high percentage of dsSDF (Table 2 and Figure 2).

### Conclusion

The results support the fact that single stranded DNA damage allows to predict the fertilisation potential, and suggest that double stranded DNA damage is related to the risk of undergoing a male-factor associated miscarriage, possibly due to a possible lack of repair of sperm dsDNA breaks by the oocyte, as we have proposed in a model. For that, it would be essential to have good quality oocytes on couples where the male show this low ssSDF and high dsSDF profile. The establishment of the 77.5% SDF threshold for neutral Comet assay offers an opportunity for idiopathic RPL without female factor patients to be diagnosed. Finally, PFGE treatments with DNase in sperm showed 48 kb bands, suggesting that the dsDNA breaks are being produced in MAR regions, which are known to be DNase sensitive. Finally, the analysis of high dsSDF fertile donors and RPL samples pointed out that non-oxidative dependent enzymatic activity could be producing the double stranded breaks detected by neutral Comet assay in these donors and patients. In this sense, the research on different strategies of sperm selection to reduce the dsSDF could improve the miscarriage rates in these patients.
